# Establishment and Characterization of a HER2-Positive Cell Line Derived From the Pleural Effusion of a Drug-Resistant Breast Cancer Patient

**DOI:** 10.3389/fcell.2021.680968

**Published:** 2021-06-01

**Authors:** Zhaoqing Li, Wenying Zhuo, Lini Chen, Xun Zhang, Cong Chen, Dengdi Hu, Yongxia Chen, Jingjing Yang, Yulu Zhou, Misha Mao, Ling Xu, Siwei Ju, Jun Shen, Qinchuan Wang, Minjun Dong, Shuduo Xie, Jichun Zhou, Linbo Wang

**Affiliations:** ^1^Key Laboratory of Cancer Prevention and Intervention, The Second Affiliated Hospital, School of Medicine, Cancer Institute, China National Ministry of Education, Zhejiang University, Hangzhou, China; ^2^Sir Run Run Shaw Hospital, Zhejiang University, Hangzhou, China; ^3^Key Laboratory of Biotherapy of Zhejiang Province, Biomedical Research Center, Hangzhou, China; ^4^The Affiliated Cixi Hospital, Wenzhou Medical University, Ningbo, China

**Keywords:** breast cancer cell line 4Z-B-1, HER2-positive, invasive ductal breast carcinoma, metastatic, HER2 targeting, phenotype change

## Abstract

Drug resistance is a daunting challenge in the treatment of breast cancer, making it an urgent problem to solve in studies. Cell lines are important tools in basic and preclinical studies; however, few breast cell lines from drug-resistant patients are available. Herein, we established a novel HER2-positive breast cancer cell line from the pleural effusion of a drug-resistant metastatic breast cancer patient. This cell line has potent proliferative capability and tumorigenicity in nude mice but weak invasive and colony-forming capability. The molecular subtype of the cell line and its sensitivity to chemotherapeutics and HER2-targeting agents are different from those of its origin, suggesting that the phenotype changes between the primary and metastatic forms of breast cancer.

## Introduction

Breast cancer, the most common malignant tumor in women, causes half a million deaths worldwide per year (Harbeck and Gnant, 2017). Human epidermal growth factor receptor 2 (HER2)-positive breast cancer accounts for approximately 15–20% of all breast cancers, and its prognosis has been improved in the past two decades by new treatments such as trastuzumab, pertuzumab and lapatinib (Loibl and Gianni, 2017). However, high rates of *de novo* resistance to trastuzumab are common in HER2-positive breast cancer in both metastatic and adjuvant settings (Martin-Castillo et al., 2013). Thus, it is vital to explore and overcome the mechanism causing resistance.

Cell lines are important tools in basic and preclinical studies, as they are convenient to maintain and allow researchers to detect the molecular mechanisms of carcinogenesis and drug resistance. Most cell lines used in studies have been maintained for decades, and they are prone to genotypic and phenotypic drifts during continuous passage (Osborne et al., 1987; Burdall et al., 2003), and few of them are from drug-resistant patients. Moreover, these cell lines mostly originate from Caucasian or African-American patients (Hackett et al., 1977; Lasfargues et al., 1978; Langlois et al., 1979; Tomlinson et al., 1998). Thus, it is necessary to establish new cell lines from Asian patients to provide a more precise *in vitro* model for preclinical studies in Asian patients.

Therefore, we established a novel HER2-positive breast cancer cell line derived from the pleural effusion of a metastatic breast cancer patient with cancer resistant to HER2-targeting therapy. In addition, we systemically characterized the cell line in terms of cell morphology, proliferation, migration, therapeutic sensitivity, protein expression, karyotype, short tandem repeat (STR) markers, and tumorigenicity.

## Materials and Methods

### Patient History

The patient (a 69-year-old Chinese female) was admitted to Sir Run Run Shaw Hospital affiliated with the Zhejiang University School of Medicine in December 2015. She complained of a lump in her right breast. A needle biopsy confirmed invasive estrogen receptor (ER) α (>90%+)-, progesterone receptor (PR) (>90%+)-, HER-2 (3+)- and Ki-67 (50%+)-positive cancer. After neoadjuvant chemotherapy (EC-TH), the patient underwent a modified radical mastectomy. The final pathological diagnosis was grade 3 invasive ductal carcinoma with ERα (70%+), PR (2–3%+), HER-2 (3+), Ki-67 (20%+), E-cadherin (+), P120 (+), cytokeratin (CK) 5/6 (−), and epidermal growth factor receptor (EGFR) (−). Axillary lymph nodes were present (6+/12). HER2-targeting treatment (trastuzumab) was administered for 1 year after surgery. Two years later, the patient was admitted to Sir Run Run Shaw Hospital for lung metastasis with pleural effusion. The pleural effusion was collected by thoracocentesis and used for cancer cell isolation and cell line establishment. The patient received HER2-targeting (trastuzumab + pertuzumab) therapy, chemotherapy, and endocrine therapy, but the disease progressed with a short PFS interval (3 months).

### Cell Lines and Reagents

SK-BR-3, MDA-MB-453, HBL-100, MCF-7, T-47D, BT-474, MDA-MB-231, BT-549, MDA-MB-468, HCC1937, and HS-578-T cell lines were purchased from the American Type Culture Collection (ATCC). HBL-100 and SK-BR-3 cells were maintained in McCoy’s 5a modified medium. MCF-7 cells were maintained in Eagle’s minimum essential medium supplemented with 0.01 mg/ml insulin. HCC-1937, T-47D, and BT-549 cells were maintained in RPMI-1640 medium supplemented with 0.023 U/ml and 0.2 U/ml insulin. MDA-MB-231, MDA-MB-453 and MDA-MB-468 cells were maintained in Leibovitz’s L-15 medium. BT-474 cells were maintained in Hybri-Care Medium supplemented with 1.5 g/L sodium bicarbonate. HS-578-T cells were maintained in DMEM supplemented with 0.01 mg/ml insulin. HER2 positive cell lines ZJU-0725, ZJU-1127 and ZJU-0327 were established by us and identified as basal like HER2 positive breast cancer cell lines and were maintained in RPMI 1640 medium. The ZJU-0725, ZJU-1127 and ZJU-0327 cell lines showed high invasiveness and potent resistance to chemotherapy in our previous study (Zhou et al., 2018). All cells were cultured in medium supplemented with 10% FBS in a humidified incubator at 37°C. MDA-MB-231, MDA-MB-453 and MDA-MB-468 cells were maintained in room air, while the other cell lines were maintained in 5% CO_2_.

### Cell Isolation and Cell Line Establishment

Once collected, the pleural effusion was transported to the laboratory on ice and centrifuged at 900 × g for 5 min. The supernatant was removed, and the cell pellet was washed with PBS with 100 U/ml penicillin and 100 μg/ml streptomycin 3 times. The cell pellet was resuspended in DMEM supplemented with 10% FBS and incubated at 37°C in 5% CO_2_. After 48 h, floating cells were transferred. Cancer cells were harvested by digestion with 0.25% trypsin–EDTA (Invitrogen) and subcultured.

### Scanning Electron Microscopy (SEM) and Transmission Electron Microscopy (TEM) Examination

For SEM, mammospheres formed in low attachment 6-well plates (Corning) or cells growing on cover glass were fixed in 2.5% glutaraldehyde (Sigma) in 0.1 M cacodylate buffer (Sigma) (pH 7.3) supplemented with 2% sucrose (Sigma) at room temperature for 1 h. The cells were washed once with cacodylate buffer and fixed with 1% osmium tetroxide in cacodylate buffer for 90 min. After being dehydrated through graded ethanol solutions, the sample cells were dried, coated with gold with an ACE200 coating system (Leica) and imaged under a Nova Nano 450 microscope (Thermo FEI).

For TEM, mammospheres were fixed with 2.5% glutaraldehyde at room temperature for 1 h, postfixed in 1% osmium tetroxide, dehydrated in a graded series of alcohol solutions, and embedded. Ultra-thin sections were cut, stained with uranyl acetate and lead citrate, and visualized under a Tecnai G2 spirit microscope (Thermo FEI).

### Evaluation of Culture Purity, Cell Cycle Distribution, and Expression of CD24/CD44 by Flow Cytometry

To evaluate cell purity, cells were fixed for 15 min in 4% formaldehyde, permeabilized in precooled 90% methanol for 10 min, blocked in 10% goat serum, and then incubated with an anti-cytokeratin (pan)-fluorescein isothiocyanate antibody (4545; Cell Signaling Technology, CST) diluted with PBS supplemented with 0.5% bovine serum albumin (incubation buffer) for 1 h. The cells were incubated in Alexa Fluor^®^-conjugated secondary antibody diluted with incubation buffer for 30 min and then counted by flow cytometry on a FACScan instrument (BD Bioscience).

To analyze cell cycle distribution, cells in the exponential growth phase were processed according to the manufacturer’s instructions (Dojindo) and then sorted by flow cytometry.

To determine the expression of CD24/CD44, cells were suspended in 100 μl PBS supplemented with 10 μl PE mouse anti-human CD24 (555428; BD Biosciences) and FITC mouse anti-human CD44 (555478; BD Biosciences) antibodies. After incubation for 30 min at 4°C in the dark, the cells were examined by flow cytometry on a FACScan instrument (BD Bioscience).

### Cell Counting Kit (CCK)-8 Cell Growth Assay

Cells were seeded in 96-well plates at a density of 2 × 10^3^ cells per well in the corresponding medium. Cells were assessed by the CCK8 (APExBio) assay after 0, 24, 48, or 72 h of incubation. The absorbance of each well at 450 nm was measured. Population doubling time (PDT) was calculated using the formula PDT = $0.693\,\text{t}/\ln\left(\frac{N\,t}{N\,0}\right)$.

### Cytogenetic Analysis

Cells were treated with 0.05 mg/ml colcemid for 6 h, incubated in 0.075 M KCl solution at 37°C for 40 min and fixed with a mixture of methanol and glacial acetic acid (3:1, v/v). Cell suspension were dropped on cold slides and stained with the Remel Giemsa Plus Stain kit (Thermo Fisher Scientific) for 10 min, and then processed by trypsin-Giemsa banding. The results are expressed according to the recommendations of the International System for Human Cytogenetic Nomenclature (ISCN) (1985).

### Mycoplasma Detection by PCR

To detect mycoplasma in cells, the culture medium of 4Z-B-1 cells at passage (P) 43 was collected, and detection was carried out according to the supplier’s instructions (Beyotime). DNA fragments were imaged under ultraviolet illumination.

### Western Blotting

Protein samples were separated via sodium dodecyl sulfate-polyacrylamide gel electrophoresis and transferred to polyvinylidene difluoride membranes (Merck Millipore). The membranes were blocked with 5% nonfat milk (BD Biosciences) in Tris-buffered saline with 0.1% Tween 20, washed, and incubated with primary antibodies against estrogen receptor (ER)-α (8644; CST), progesterone receptor A/B (PR) (8757; CST), HER2 (AB16899; Abcam), CDK6 (3524-1; EPITOMICS), CyclinE1 (1655-1; EPITOMICS), ZO1 (8193; CST), E-Cadherin (3195; CST), ZEB1 (3396; CST), N-Cadherin (13116; CST), Vimentin (5741; CST), Snail (3879; CST), Slug (9585; CST), Claudin-1 (13255; CST), β-catenin (8480; CST), EGFR (4267; CST), p-EGFR (Y1068) (3777; CST), Caveolin (3267; CST), STAT3 (bs-1141R; BIOSS), p-PTEN (S380) (9551; CST), Akt (4691; CST), p-Akt (T306)(13038; CST), Bcl-X (1018-1; EPITOMICS), Bcl-2 (0407-7; Hua Bio) and β-actin (sc-477748; Santa Cruz). The membrane was incubated with diluted horseradish peroxidase (HRP)-conjugated secondary antibody (1:2000, CST), treated with a Pico ECL kit (FDbio) and imaged with an Amersham Imager 600 (GE Healthcare).

### Evaluating Sensitivity to Anticancer Agents

Cells were plated in 96-well plates at a density of 8 × 10^3^ cells per well and then treated with anticancer agents at the appropriate concentrations for 48 h or 72 h. After incubation, viability was analyzed by the CCK-8 assay. The absorbance of each well at 450 nm was measured, and survival rate curves were plotted.

### *In vitro* Invasion Assay

For transwell assays, 4Z-B-1 at P53 or SK-BR-3 cells (5 × 10^4^ cells/well) were seeded in serum-free medium in transwell inserts (Corning) either coated with Matrigel (BD Biosciences) or left uncoated. The receiver plates were filled with medium containing 20% FBS. After incubation for 16 h, the cells that had penetrated through the pores were fixed with 4% paraformaldehyde (Solarbio) and stained with 0.1% crystal violet (Solarbio). The cells were then washed with PBS and viewed under an inverted microscope (ZEISS).

A 3D spheroid BME cell invasion assay was conducted using a kit (3500-096-K; Trevigen) according to the supplier’s instruments. The spheroid in each well was photographed every 24 h using a 4 × objective (ZEISS). Images were analyzed with ImageJ.

### Colony-Forming Assay

To assess colony-forming capacity, 2000 cells were seeded in 6-well plates per well in the corresponding medium, and the medium was replaced as necessary. After 7 days, the cells were washed with PBS 3 times, fixed in 4% paraformaldehyde (Solarbio) and stained with 0.1% crystal violet (Solarbio). Colonies were counted, and the results were analyzed.

### Tumorigenicity in Nude Mice

A total of 4 × 10^6^ 4Z-B-1 cells at P50 resuspended in a 0.1 ml mixture of PBS and Matrigel (Corning) were subcutaneously injected into each nude mouse (BALB/C nu; 4 weeks old; SLAC Laboratory Animals Company). The animals were maintained in laminar flow cabinets under specific pathogen-free conditions. The volume of the tumors and the weights of the mice were recorded every 3 days. After 21 days, tumor tissue was collected, measured and weighed, fixed in 10% formalin, embedded in paraffin, and processed for hematoxylin and eosin (H&E) and immunohistochemical (IHC) staining.

### H&E and IHC Staining

Paraffin-embedded specimens were cut into 4-μm-thick sections and were mounted on polylysine-coated slides, deparaffinized in xylene, rehydrated in a graded series of alcohol solutions, and stained with H&E. For immunohistochemical analysis, the slides were heated in 10 mM sodium citrate (pH 6.5) with a pressure cooker for 10 min. After being treated with 3% H_2_O_2_ for 5 min and blocked with 10% normal goat serum for 30 min, the sections were probed with primary antibodies against ER-α, PR, HER2, E-cadherin, CK5/6, EGFR, P120, or Ki-67 (Dako) for 1 h at room temperature. Detection of the primary antibody and color development were performed using the GT vision III Immunohistochemical Assay Kit (GK500710; Gene Tech) according the manufacturer’s protocol. The sections were counterstained with hematoxylin.

### Verification of Cell Lines by ATCC

The 4Z-B-1 cell line at P60 was authenticated by ATCC (ATCC sales order no. SO0546653). Seventeen STR loci plus the sex-determining locus amelogenin were amplified using the commercially available PowerPlex^®^ 18D Kit from Promega. The cell line sample was processed using the ABI Prism^®^ 3500xl Genetic Analyzer. The data were analyzed using GeneMapper^®^ ID-X v1.2 software (Applied Biosystems). Appropriate positive and negative controls were run and confirmed. The 4Z-B-1 cell line was not a match to any cell line in the ATCC, DSMZ or ExPASy databases.

### Statistical Analysis

The data of at least three independent experiments were presented as the mean ± standard deviation, and analyzed using Prism software (GraphPad Inc.). Differences with a *P* value of < 0.05 were regarded statistically significant.

## Results

### Morphological Characterization

**FIGURE 1:**
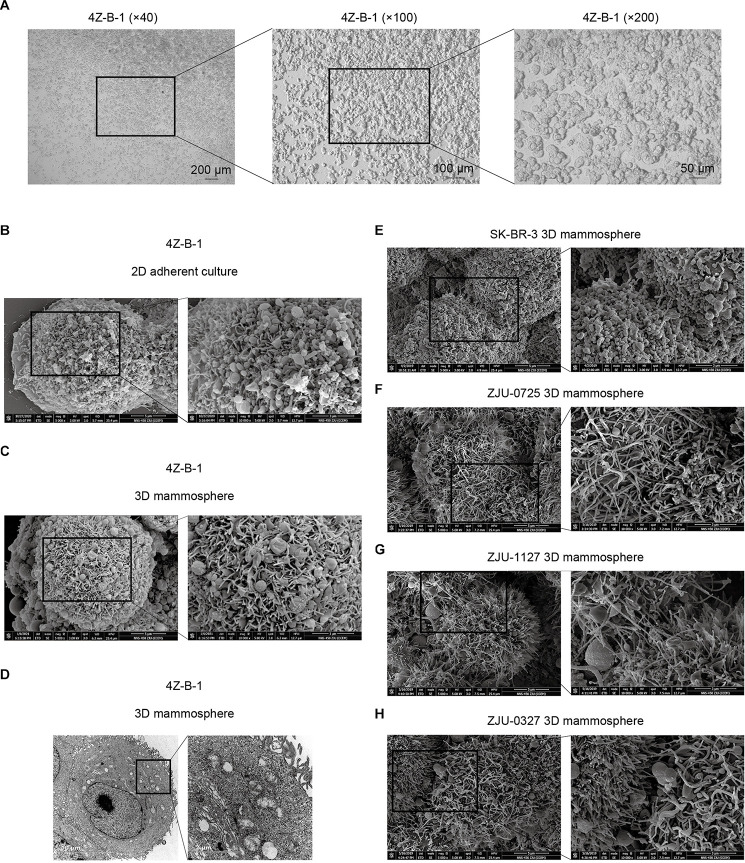
Morphological analysis of the 4Z-B-1 cell line. **(A)** 4Z-B-1 cells were round and piled up when grown at high density. **(B,C)** Projections of both spherical and finger-like extensions could be observed on the surface of both 2D adherently cultured 4Z-B-1 cells and 3D 4Z-B-1 mammospheres under scanning electron microscopy (SEM). **(D)** Nuclei and abundant organelles could be observed under TEM. **(E)** The projections on the surface of SK-BR-3 cells were almost spherical. **(F–H)** The projections on the surface of ZJU-0725, ZJU-1127, and ZJU-0327 cells were mostly finger-like. Magnification, **(A)** left, ×40; middle, ×100; right, ×200; **(B,C,E–H)**: left, ×5000; right, ×10000; **(D)** left, ×4800; right, ×18500.

The 4Z-B-1 cell line at P60 was deposited in the China Center for Type Culture Collection (CCTCC) (No. C202123), and the origin information for 4Z-B-1 cells was listed in Table 1. The 4Z-B-1 cells (at P46) were round in shape with poorly differentiated morphology (Figure 1A). When grown at high density, the cells piled up, showing a loss of contact inhibition, indicating cell malignancy.

**TABLE 1:** Origin information of the 4Z-B-1 cell line.

**General feature**	**Description**
Animal	Human
Genus	Homo
Species	Sapiens
Gender	Female
Age at sampling	69 years
Tissue derived	Pleural effusion
Case history	Breast cancer

When examined via SEM, projection of both spherical and finger-like extensions could be observed on the surface of both 2D adherently cultured cells and 3D mammospheres of 4Z-B-1 (at P46) (Figures 1B,C). In contrast, the projections on the surface of SK-BR-3 (Figure 1E) and 3 HER2-positive cell lines we established previously (Zhou et al., 2018), namely, ZJU-0725, ZJU-1127 and ZJU-0327, were almost spherical-like and finger-like, respectively (Figures 1F–H). When observed under TEM (at P46) (Figure 1D), large and irregular nuclei with a sunken nuclear membrane and abundant organelles, including rough and smooth endoplasmic reticula, mitochondria, polyribosomes and Golgi apparatus, could be observed.

### Purity and Mycoplasma Contamination

**FIGURE 2:**
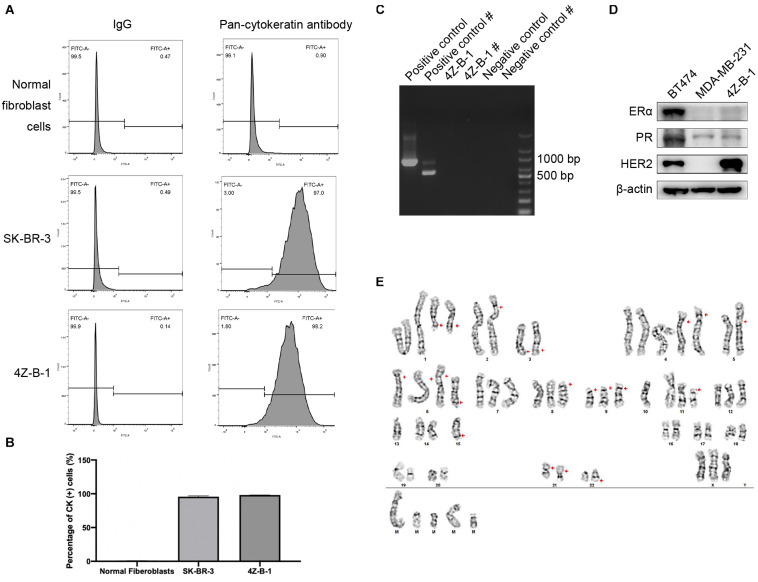
Purity, mycoplasma contamination, molecular subtype and karyotype analysis of 4Z-B-1 **(A,B)** The purity of the normal fibroblast cells, SK-BR-3 and 4Z-B-1 was evaluated, and the expression of pan-cytokeratin was detected by flow cytometry. **(C)** 4Z-B-1 was detected free of mycoplasma. # represents the second PCR, which had improved sensitivity and specificity. **(D)** 4Z-B-1 cells were negative for ERα and PR but positive for HER2. **(E)** Representative karyotype of 4Z-B-1 cells: 61, XXX; red arrows point to +del(1)(q21) × 2, –2, add(2)(p25), –3, del(3)(q21), +4 × 2, –5, add(5)(p15.3), add(6)(p25) × 3, +del(6)(q23), del(8)(p11.2), del(9)(p12) × 3, –10 × 2, del(11)(p11.2), –13 × 2, –14, –15 × 2, der(15)t(15:?)(q26.1;?), –16, –17, –18, –19, –20, –21, add(21)(p12) × 2, –22, der(22)t(22;?)(q13;?), +mar1, +mar2, +mar3, mar4, and +mar5 [cp5].

Pan-CK are widely used epithelial markers (Moll et al., 1982; Hass and Bertram, 2009; Blanchette-Farra et al., 2018). The purity of 4Z-B-1 cells was 98.2%, as assessed at P45 by flow cytometry analysis of the expression of pan-CK (Figures 2A,B). The expression rates of pan-CK were 0.9% and 97.0% in normal fibroblast cells and SK-BR-3 cells, respectively, which were used as negative and positive controls, respectively. 4Z-B-1 cells (at P43) were found to be free of mycoplasma contamination (Figure 2C).

### Molecular Phenotype and Chromosome Analysis

To analyze the molecular phenotype of 4Z-B-1 cells, the expression of ERα, PR and HER2 in 4Z-B-1 cell at P48 was assessed by Western blotting with BT-474 (triple-positive subtype) and MDA-MB-231 (triple-negative subtype) as controls. As shown in Figure 2D, the expression levels of ERα and PR in 4Z-B-1 cells were similar to those in MDA-MB-231 cells, and the expression level of HER2 was even higher than that in BT-474 cells. Thus, we identified the 4Z-B-1 cell line as a HER2-positive breast cancer cell line.

Karyotype analysis revealed that 4Z-B-1 (at P51) was nearly triploid, with the number of chromosomes in the range of 61-72. Trypsin-Giemsa banding revealed numerous chromosomal deletions and additions (Figure 2D).

### Proliferative, Migratory, and Invasive Potential

**FIGURE 3:**
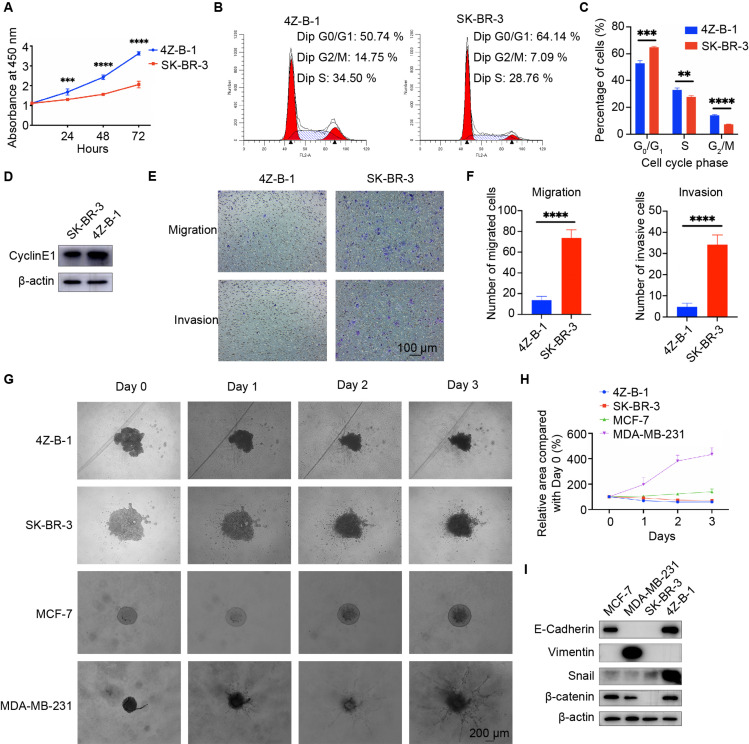
Proliferative, migratory and invasive potential of the 4Z-B-1 cell line. **(A)** Growth curves of 4Z-B-1 and SK-BR-3 cells determined by the CCK8 assay. **(B,C)** Distribution of 4Z-B-1 and SK-BR-3 cells determined by flow cytometry and statistical analysis. **(D)** Expression of CyclinE1 in 4Z-B-1 and SK-BR-3 cells assessed by Western blotting. **(E,F)** Representative micrographs and statistical analysis of migrated and invasive 4Z-B-1 and SK-BR-3 cells. **(G,H)** Representative micrographs and statistical analysis of the invasive area of 4Z-B-1, SK-BR-3, MCF-7, and MDA-MB-231 mammospheres in Matrigel. **(I)** Expression of EMT markers in 4Z-B-1, SK-BR-3, MCF-7, and MDA-MB-231 cells determined by Western blotting. ****p < 0.0001. Magnification, **(E)** ×100; **(G)** ×40.

The proliferative potential of 4Z-B-1 cells at P55 was assessed via the CCK-8 assay, and SK-BR-3 was used as a control. As shown in Figure 3A, 4Z-B-1 cells grew faster than SK-BR-3 cells, with a PDT of 40 h. The DNA content of 4Z-B-1 (at P56) and SK-BR-3 cells was determined by flow cytometry, and the distribution of the cell phases was analyzed (Figures 3B,C). Compared with SK-BR-3 cells, 4Z-B-1 cells were distributed more in S phase and G2/M phase and less in G0/G1 phase. Western blotting assays also showed that the expression level of CyclinE1 was higher in 4Z-B-1 cells than SK-BR-3 cells (Figure 3D).

However, in contrast to the rapid growth rate of 4Z-B-1 cells, the migratory and invasive potential of these cells (at P58) was much weaker than those of SK-BR-3 cells, as determined by the transwell assay (Figures 3E,F). A 3D spheroid BME cell invasion assay was also performed to test the invasive potential of 4Z-B-1 cells (at P59) in a 3D model. SK-BR3, MCF-7 and MDA-MB-231 were used for comparison. 4Z-B-1, SK-BR-3 and MCF-7 cells remained as cell aggregates and did not invade the surrounding matrix, while MDA-MB-231 cells invaded the surrounding matrix with spindle-like protrusions (Figures 3G,H). The expression levels of epithelial-mesenchymal transition (EMT) markers in these cells were determined by Western blotting (Figure 3I). 4Z-B-1 cells expressed high levels of E-cadherin and low levels of vimentin. Snail and β-catenin were also highly expressed in 4Z-B-1 cells.

### Sensitivity to HER2 Targeting Agents

**FIGURE 4:**
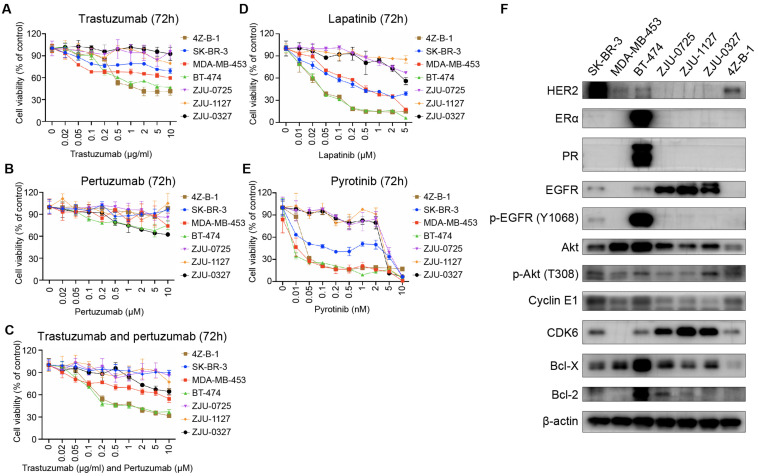
Sensitivity to HER2-targeting agents and expression of associated proteins. **(A–E)** Comparison of sensitivity to trastuzumab, pertuzumab, the combination of trastuzumab and pertuzumab, lapatinib and pyrotinib between the 4Z-B-1 cell line and other HER2-positive breast cancer cell lines. **(F)** Expression of HER2-targeting agent sensitivity-associated genes in the 4Z-B-1 cell line and other HER2-positive breast cancer cell lines.

Since 4Z-B-1 cells were identified as HER2-positive cells and originated from a Herceptin-resistant patient, we tested the sensitivity of the cells to HER2-targeting agents, including trastuzumab, pertuzumab, lapatinib, pyrotinib and a combination of trastuzumab and pertuzumab. The sensitivity of several other HER2-positive breast cancer cell lines, including SK-BR-3, MDA-MB-453, BT-474, ZJU-0725, ZJU-1127, and ZJU-0327, was also tested for comparison. 4Z-B-1 cells (at P63) showed sensitivity to trastuzumab and the combinations of trastuzumab and pertuzumab and lapatinib and pyrotinib but showed resistance to pertuzumab alone (Figures 4A–E). In contrast, the ZJU-0725, ZJU-1127, and ZJU-0327 cell lines, which were identified as basal-like HER2-positive breast cancer cell lines, showed resistance to all of these HER2-targeting agents (Figures 4A–E).

According to previous studies, promotion of HER2 downstream signaling pathways such as PI3K/Akt, activation of alternate receptor pathways such as EGFR, crosstalk between estrogen receptor and HER2, and alterations in apoptosis and cell cycle control contribute to breast cancer resistance to trastuzumab (Pohlmann et al., 2009; Wong and Lee, 2012; Luque-Cabal et al., 2016). To explain the difference in sensitivity to HER2 targeting agents between these HER2-positive cell lines, the expression and activation of associated genes were determined by Western blotting. As shown in Figure 4F, the expression of ERα, PR, EGFR, p-EGFR (Y1068), Akt, CDK6, Bcl-X and Bcl-2 was low in 4Z-B-1 cells, which is consistent with the sensitivity of these cells to HER2-targeting agents. In contrast, the expression of EGFR and CDK6 in the resistant cell lines ZJU-0725, ZJU-1127 and ZJU-0327 was much higher than that in the other cell lines.

### Sensitivity to Other Anticancer Agents

**FIGURE 5:**
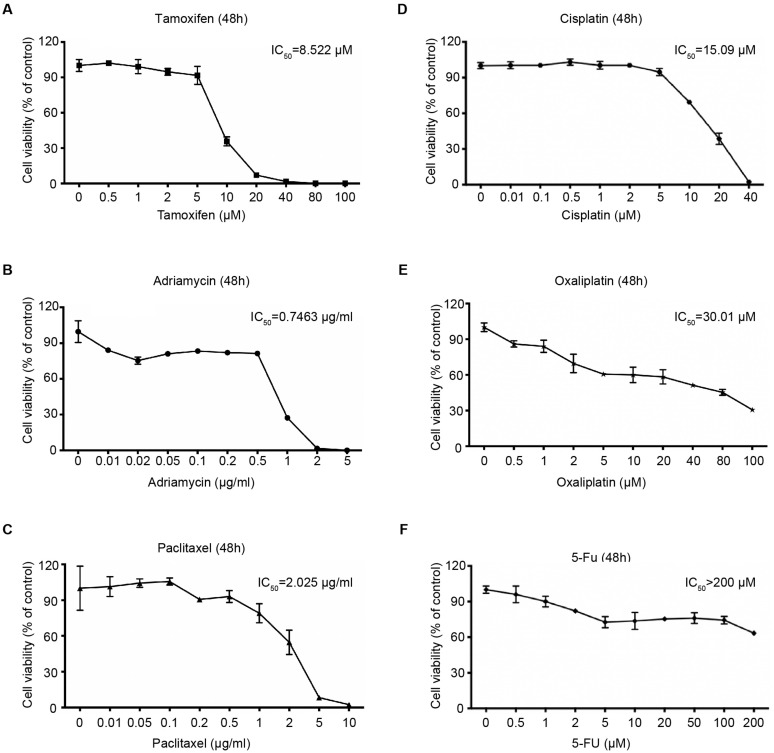
Sensitivity of the 4Z-B-1 cell line to other anticancer agents. **(A–F)** Sensitivity of 4Z-B-1 cells to tamoxifen, adriamycin, paclitaxel, cisplatin, and oxaliplatin and 5-Fu.

We also determined the sensitivity of 4Z-B-1 (at P64) to several other agents that are commonly used in cancer treatment. As shown in Figures 5A–F, tamoxifen, adriamycin, paclitaxel, cisplatin and oxaliplatin killed 4Z-B-1 cells efficiently with IC_50_ values of 8.522 μM, 0.7463 μg/ml, 2.025 μg/ml, 15.09 μM and 30.01 μM, respectively, while 5-Fu seemed to be harmless to 4Z-B-1 cells, with an IC_50_ higher than 200 μM.

### Gene Expression Analysis

**FIGURE 6:**
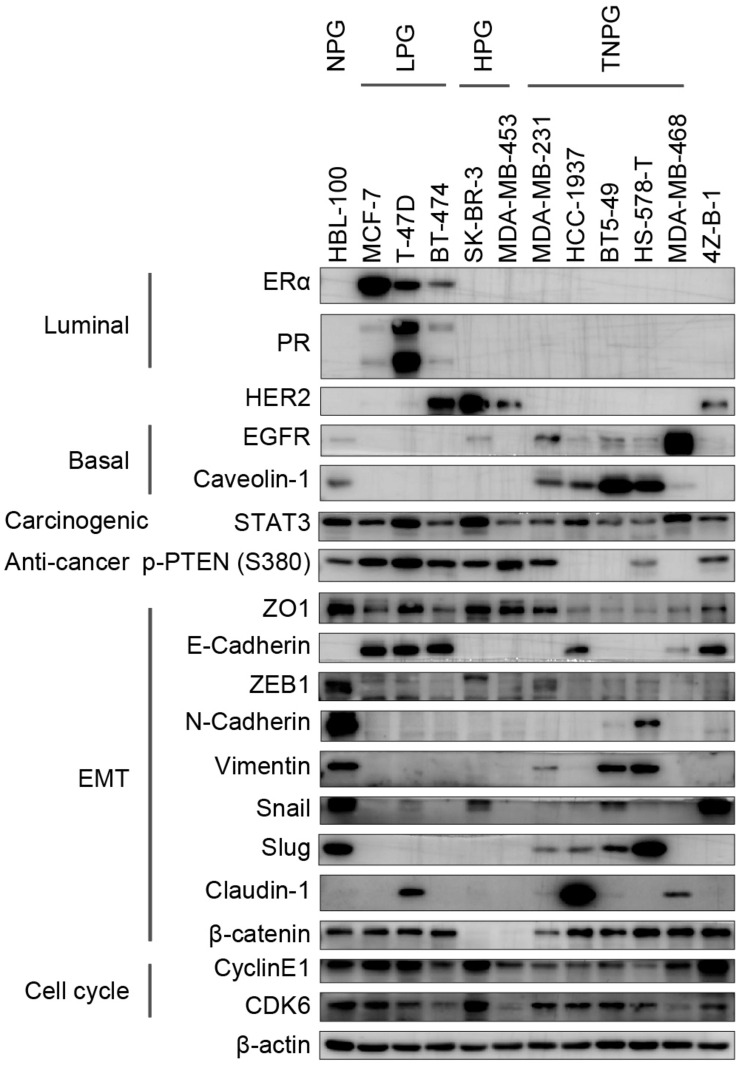
Gene expression analysis of the 4Z-B-1 cell line. Protein expression pattern in the 4Z-B-1 cell line compared with the normal, luminal, HER2-positive and triple-negative phenotype breast cancer cell line groups.

Western blotting was applied to characterize the gene expression patterns of 4Z-B-1 cells (at P65) compared with those of other breast cancer cell lines, which were divided into the normal phenotype group (NPG), luminal phenotype group (LPG), HER2 positive phenotype group (HPG), and triple-negative phenotype group (TNPG). As shown in Figure 6, 4Z-B-1 cells were negative for luminal markers (ERα and PR) and basal markers (EGFR and caveolin-1) but positive for HER2. The expression of the carcinogenic marker STAT3 and the anticancer marker p-PTEN (S380) was moderate. 4Z-B-1 cells were positive for the epithelial markers ZO1 and E-cadherin and negative for most mesenchymal markers except for snail and β-catenin. 4Z-B-1 cells expressed relatively high levels of cyclin E1 and CDK6, which is consistent with the strong proliferative ability of these cells.

### Putative Stemness and Tumorigenicity *in vitro* and *in vivo*

**FIGURE 7:**
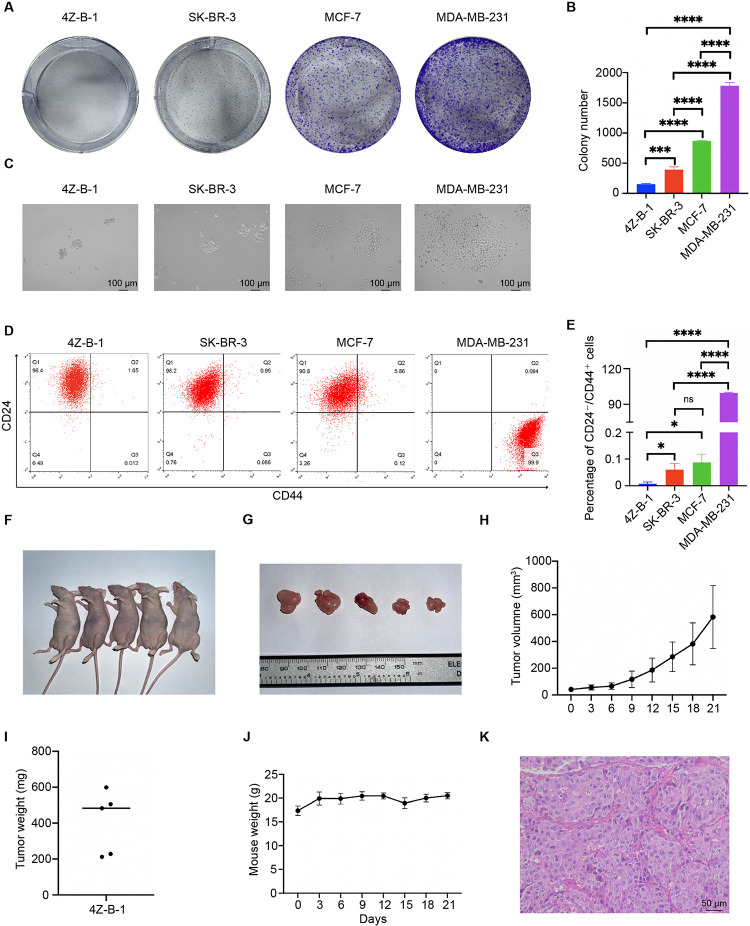
Colony-forming capability, putative stemness and tumorigenicity in immunodeficient mice. **(A,B)** Colony-forming capability of the 4Z-B-1, SK-BR-3, MCF-7, and MDA-MB-231 cell lines and statistical analysis. **(C)** Morphology of colonies formed by 4Z-B-1, SK-BR-3, MCF-7, and MDA-MB-231 cells. **(D,E)** Percentage of CD24-/CD44+ cells among 4Z-B-1, SK-BR-3, MCF-7 and MDA-MB-231 cells and statistical analysis. **(F,G)** Tumor formation in immunodeficient mice (tumor size indicated by the ruler). **(H)** Tumor growth curves of 4Z-B-1 cells. **(I)** Weight of 4Z-B-1 cell xenografts. **(J)** Weight curve of the experimental mice. **(K)** H&E staining of 4Z-B-1 cells xenografts. ns, *p* > 0.05; *p < 0.05; ***p < 0.001; ****p < 0.0001. Magnification, **(C)** ×100; **(K)** ×200.

A colony-forming assay was applied to assess the tumorigenicity of 4Z-B-1 cells *in vitro*. Although 4Z-B-1 cells exhibited potent proliferative ability, as shown above, their ability to form colonies was very weak (Figures 7A,B). 4Z-B-1 (at P52) formed fewer colonies than SK-BR-3 cells. On the other hand, the colonies formed by 4Z-B-1 cells were quite different in morphology from those formed by SK-BR-3, MCF-7, or MDA-MB-231 cells. The 4Z-B-1 cells piled up to form a three-dimensional and dense structure, while other cell lines formed flat and loose structures (Figure 7C).

Since stemness is related to the tumorigenicity of cells (Li et al., 2017; Shamsian et al., 2020), we assessed putative stemness by assessing the expression of CD24 and CD44. Few CD24^–^/CD44^+^ cells were detected by flow cytometry in the 4Z-B-1 (at P54), SK-BR-3 and MCF-7 cells. In contrast, MDA-MB-231 cells were mostly CD24^–^/CD44^+^ (Figures 7D,E).

Xenograft transplantation was carried out to assess the *in vivo* tumorigenicity of 4Z-B-1 cells. All mice developed tumors after subcutaneous implantation of 4Z-B-1 cells, and the tumors grew rapidly (Figures 7F–I). The weight of the mice did not vary significantly (Figure 7J). H&E staining revealed that 4Z-B-1 cells developed histological high-grade adenocarcinoma (Figure 7K). Neither lymph node metastasis nor distant metastasis was observed.

### Immunohistochemical Analysis of Original Breast Tumors and Xenografts

**FIGURE 8:**
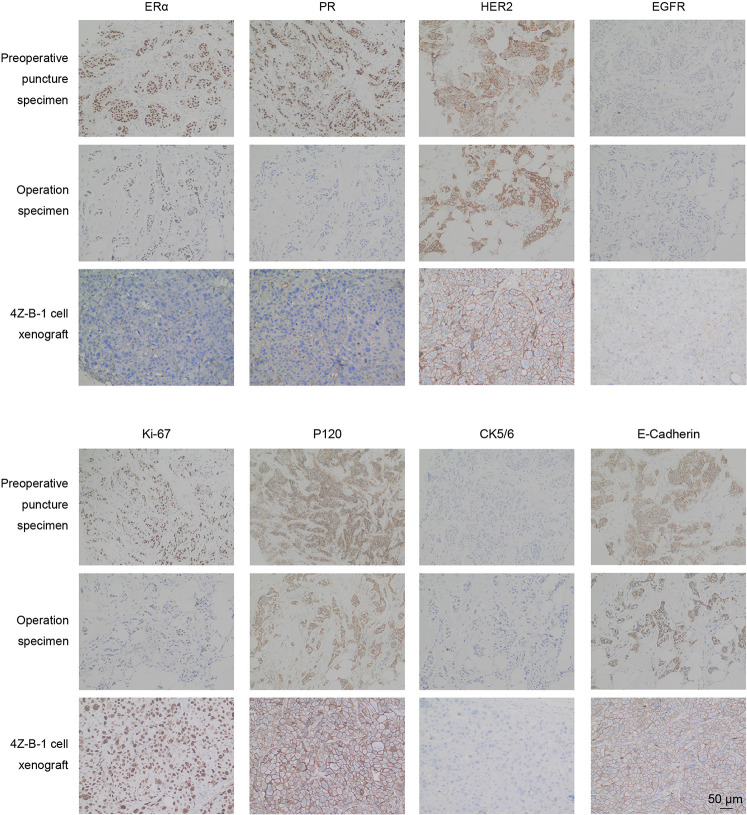
IHC analysis of original and xenografted tumors. The expression of ERα and PR was decreased in 4Z-B-1 cell xenografts, while that of EGFR and Ki-67 was elevated. The expression of HER2, P120, CK5/6, and E-cadherin was similar in preoperative puncture specimens, operation specimens and 4Z-B-1 xenografts. Magnification: ×200.

Immunohistochemical analysis was applied to compare the expression of ERα, PR, HER2, EGFR, Ki-67, P120, CK5/6 and E-cadherin in preoperative puncture specimens, operation specimens and 4Z-B-1 xenografts (Figure 8). The expression of ERα and PR was decreased in operation specimens compared with preoperative core needle biopsy specimens and absent in 4Z-B-1 cell xenografts. In contrast, HER2 was strongly positive in all three samples. EGFR was negative in preoperative puncture specimens and operation specimens but weakly positive in 4Z-B-1 cell xenografts. Ki-67 expression was low in operation specimens compared with preoperative core needle biopsy specimens but relatively high in 4Z-B-1 cell xenografts. The expression of P120, CK5/6, and E-cadherin was similar in the three samples.

### DNA Fingerprinting/STR Analysis

STR analysis of the 4Z-B-1 cell line confirmed that it was of human origin and was distinct from other cell lines in the ATCC and DSMZ databases (ATCC ID No. STRB3244) (Table 2).

**TABLE 2:** STR profile of the 4Z-B-1 cell line.

**STR locus**	**4Z-B-1**
TH01	7, 9
D5S818	11, 13
D13S317	9
D7S820	9, 10
D16S539	10
CSF1PO	10
Amelogenin locus	X
vWA	14, 16
TPOX	8, 11

*CSF1PO, human c-fms proto-oncogene for CSF-1 receptor gene; STR, short tandem repeat; TPOX, intron 10 of the human thyroid peroxidase gene; vWA, intron 40 of the von Willebrand factor.*

## Discussion

In this study, we isolated a HER2-positive breast cancer cell line from the pleural effusion of a breast cancer patient with lung and pleural metastasis and named it 4Z-B-1. A series of experiments were carried out to characterize the novel cell line. The cell line was passaged for more than 2 years and subcultured for more than 100 passages without obvious changes in morphology or proliferative potential after cryopreservation and resuscitation.

Morphologically, 4Z-B-1 cells were round and poorly differentiated. As the cells divided, they piled up to form dense 3D dense structures (Figures 1A, 6B). This implied loss of contact inhibition and cell malignancy, and the formation of clusters may increase the metastatic potential of the cells (Gkountela et al., 2019). Under SEM, projections of both spherical and finger-like extensions could be observed on the surface of 4Z-B-1 cells. Shurer et al. proposed that these external extensions are generated by the synergy between processes in the cell interior and the external glycocalyxes, which are composed of mucin biopolymers and long-chain polysaccharides (Shurer et al., 2019). They also demonstrated that these structures are associated with plasma membrane instabilities and the secretion of extracellular vesicles. Thus, the differences in morphology between the cell lines suggested various different cell phenotypes.

The 4Z-B-1 cell line originated from a patient resistant to Herceptin but turned out to be sensitive to HER2-targeting agents as a cell line *in vitro* (Figures 4A–E). This may reflect the importance of the tumor microenvironment in the therapeutic resistance of tumors (Qu et al., 2019). On the other hand, compared with cell lines relatively resistant to HER2-targeting agents, the 4Z-B-1 cells expressed lower levels of EGFR and CDK6, which is consistent with studies suggesting that EGFR and CDK6 play an important role in HER2-targeting agent resistance (Gallardo et al., 2012; Goel et al., 2016).

Few 4Z-B-1 cells were CD24^–^/CD44^+^ (Figures 7D,E), reflecting the cell line’s low putative stemness as demonstrated by a colony-forming assay (Figures 7A,B). However, 4Z-B-1 cells were highly tumorigenic in nude mice (Figures 7F–I). We noticed that the molecular subtype of the cells changed during metastasis. The cell line was ERα-negative and PR-negative, while the primary specimen from the patient was ERα-positive and PR-positive (Figure 8). Subtype conversion in metastases is a common phenomenon in breast cancer (Cejalvo et al., 2017; Hulsbergen et al., 2020). It has been reported that 30% of luminal B breast cancer tumors convert to different subtypes in metastatic foci, and 14.3% of luminal A and B breast cancer tumors convert to HER2-enriched tumors (Cejalvo et al., 2017). Acquisition of a luminal B- or HER2-enriched profile by luminal/HER2-negative tumors during metastatic progression may be related to tumor evolution or acquisition of estrogen independence (Cejalvo et al., 2017).

## Conclusion

In conclusion, we established a novel HER2-positive breast cancer cell line from the pleural effusion of a human breast cancer patient. This cell line exhibited unique characteristics, and its phenotype was different from that of its origin. This novel cell line could serve as a new model for the investigation of metastatic HER2-positive breast cancer both *in vitro* and *in vivo*.

## Data Availability Statement

The original contributions presented in the study are included in the article/supplementary material, further inquiries can be directed to the corresponding authors.

## Ethics Statement

The studies involving human participants were reviewed and approved by Institutional Review Board of Affiliated Sir Run Run Shaw Hospital, Zhejiang University. The patients/participants provided their written informed consent to participate in this study. The animal study was reviewed and approved by Institutional Review Board of Affiliated Sir Run Run Shaw Hospital, Zhejiang University. Written informed consent was obtained from the individual(s) for the publication of any potentially identifiable images or data included in this article.

## Author Contributions

LW, JZ, and ZL designed the study. ZL, WZ, LC, and XZ performed the experiments. ZL, CC, and DH contributed to writing the manuscript. YC, JY, YZ, MM, and LX analyzed the data. SJ, JS, and QW contributed to manuscript review and revision. MD and SX provided technical support. All authors contributed to the article and approved the submitted version.

## Conflict of Interest

The authors declare that the research was conducted in the absence of any commercial or financial relationships that could be construed as a potential conflict of interest.

## Abbreviations

ATCC: American type culture collection
CK: cytokeratin
EGFR: epidermal growth factor receptor
EMT: epithelial-mesenchymal transition
HER2: human epidermal growth factor receptor 2
SEM: scanning electron microscopy
STR: short tandem repeat
TEM: transmission electron microscopy.
